# *In Utero* and Postnatal Propylthiouracil-Induced Mild Hypothyroidism Impairs Maternal Behavior in Mice

**DOI:** 10.3389/fendo.2018.00228

**Published:** 2018-05-14

**Authors:** Miski Aghnia Khairinisa, Yusuke Takatsuru, Izuki Amano, Michifumi Kokubo, Asahi Haijima, Wataru Miyazaki, Noriyuki Koibuchi

**Affiliations:** ^1^Department of Integrative Physiology, Graduate School of Medicine, Gunma University, Maebashi, Japan; ^2^Department of Pharmacology and Clinical Pharmacy, Universitas Padjadjaran, Bandung, Indonesia

**Keywords:** maternal behavior, mild hypothyroidism, cognition, thyroid hormones, hippocampus

## Abstract

Thyroid hormones (THs) play crucial roles in general and brain development. Even if the hypothyroidism is mild, it may alter brain function, resulting in irreversible behavioral alterations. Although various behavioral analyses have been conducted, the effects of propylthiouracil (PTU) treatment during *in utero* and postnatal periods on maternal behavior have not yet been studied. The present study examined in mice whether THs insufficiency during development induce behavioral changes. Pregnant C57BL/6j mice were divided into three groups, and each group was administered different dosages of PTU (0, 5, or 50 ppm) in drinking water during *in utero* and postnatal periods (from gestational day 14 to postnatal day 21). First, locomotor activity and cognitive function were assessed in the offspring at 10 weeks. Next, female offspring were mated with normal mice and they and their offspring were used to assess several aspects of maternal behavior (identifying first pup, returning all pups to nest, time spent nursing, and licking pups). As expected, locomotor and cognitive functions in these mice were disrupted in a PTU dose-dependent manner. On postpartum day 2, dams who had been exposed 50 ppm PTU during *in utero* and postnatal periods displayed a significantly longer time identifying the first pup and returning all three pups back to the nest, less time nursing, and decreased licking behavior. The decrease in maternal behavior was significantly correlated with a decrease in cognition. These results indicate that insufficiency of THs during *in utero* and postnatal periods impairs maternal behavior, which may be partly induced by impaired cognitive function.

## Introduction

Thyroid hormones (THs) (l-triiodothyronine, T3; thyroxine, T4) play crucial roles in the development and functional maintenance of the central nervous system. During development, THs regulate the growth and morphogenesis of brain and neuronal cells. In the cerebellum, for example, THs affect the dendritic growth of cerebellar Purkinje cells, the proliferation and migration of granule cells, and the synaptogenesis of cerebellar neurons (1). As a consequence, insufficiency of THs during development leads to disrupted motor coordination in adulthood (2, 3). Similarly in the hippocampus, the migration of granule cells and dendritic growth of pyramidal cells are also disrupted by hypothyroidism (4, 5), inducing aberrant synaptic function and learning (6). Increased anxiety- and depression-like behaviors in mice with hypothyroidism have also been reported (7–12). Overall, these results indicate that THs act in multiple regions of the brain during development and adulthood, affecting various behaviors throughout life.

Maternal behavior is one of the social behaviors between mother and offspring. Several hormones, including estrogen (13, 14), prolactin (15), vasopressin, and oxytocin (15–17), are involved in regulating maternal behavior. In rats, maternal behaviors, particularly licking and grooming, regulate the development of the offspring’s endocrine, emotional, and cognitive responses to stress (18). Maternal behavior tests have been used extensively as a parameter for brain development and stress responses (19). THs have also been implicated in maternal behavior by regulating hippocampal serotonin activity and glucocorticoid expression (20, 21). Recent studies have also shown that type 3 deiodinase (D3), which is a critical factor in regulating TH availability in the brain, may be also involved in controlling maternal behavior (12). D3 knockout mice showed impaired maternal behavior, increased aggression toward their offspring, and low levels of oxytocin and vasopressin with altered expression of their receptors (12). Although these results indicate that THs play an important role in controlling maternal behavior, the mechanisms of TH action on maternal behavior remain unclear.

In this study, we examined whether *in utero* and postnatal propylthiouracil (PTU)-induced mild hypothyroidism impairs maternal behavior in mice. To achieve this aim, we rendered pregnant mice hypothyroid by administering PTU in drinking water during pregnancy and the lactating period. Female offspring were used to examine the locomotor and cognitive functions using open field test, object recognition test (ORT), and object-in-location recognition test (OLT). This was followed by mating them with normal male mice and after delivery, assessing maternal behavior by examining pup retrieval characteristics, nursing, and licking times. Our study showed that even mild hypothyroidism disrupted maternal behavior and that the magnitude of disruption of maternal behavior was correlated with anxiety-like behavior and impaired memory function.

## Materials and Methods

### Animals

The animal experimentation protocol in this study was approved by the Animal Care and Experimentation Committee of Gunma University. All efforts were made to minimize the number of animals used in this study and their suffering. Mice (C57BL/6 strain) used in this study were bred in the Bioresource Center of Gunma University Graduate School of Medicine. They were kept at 24°C under a 12-h light/12-h dark cycle (light 7:00 a.m.–7:00 p.m.) and 50–70% relative humidity, with food available *ad libitum*.

### Treatment Schedule

**Figure 1:**
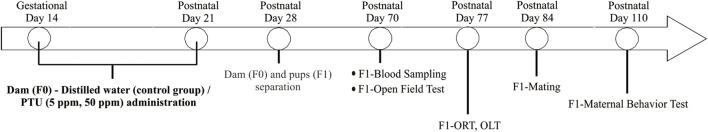
Schematic drawing of experimental procedure. Pregnant C57BL/6 mice received 5 ppm or 50 ppm propylthiouracil (PTU) from gestational day 14 to postnatal day 21. On postnatal day 28, we separated dams from pups. Some pups were sacrificed on postnatal day 70 for serum analysis, whereas others were kept for behavioral tests.

A schematic figure of the animal treatment schedule is shown in Figure 1. Adult male and female mice were mated over 1 day and the pregnancy was confirmed as gestational day 1. Dams (termed F0) were rendered hypothyroid by administrating either 5 or 50 ppm of PTU in drinking water from gestational day 14 to postpartum day 21 (22). Control mice were administered distilled water. After weaning on postpartum day 28, dams (control = 4 mice, 5 ppm = 6 mice, 50 ppm = 6 mice) and all female offspring (termed F1) (control = 12 mice, 5 ppm = 16 mice, 50 ppm = 16 mice) were separated and given distilled water only. Behavioral testing of F1 female offspring started at postnatal day 70. Treatment and individual housing continued throughout the behavioral testing period.

After behavioral tests, control and PTU-exposed female F1 mice were mated with normal male mice. Once pregnancy was suspected by abdominal enlargement, female mice were housed individually and checked every morning until delivery. Maternal behavior tests of F1 were performed at postnatal day 110. The number of mice in each experiment was described in Table S2 in Supplementary Material.

### Blood Collection and TH Measurement

Blood was collected from a subset of F1 mice at postnatal day 70 under an intraperitoneal anesthetic (i.p.) injection of ketamine/xylazine (22.5 mg/ml ketamine and 1 mg/ml xylazine in 0.9% NaCl; 5 ml/kg). Ten F1 mice from five different F0 dams were used for measurement of TH levels in each group, while the remainder participated in the behavioral studies. Blood was withdrawn from the heart. Blood samples were then centrifuged (14,000 rpm) to separate serum. Serum was kept at −80°C until use. Free T4 (fT4) and free T3 (fT3) concentrations in serum were measured by chemiluminescent micro particle immunoassay (CMIA) on a single automatic analyzer using the architect SYSTEM (Abbott ARCHITECT i2000; Abbott Laboratories, Maidenhead, United Kingdom). The lowest detectable limits were 1.00 ρg/ml and 0.40 ng/dl for fT3 and fT4, respectively (23). Five tube samples were used for measurement, and serum samples from two mice in the hypothyroidism group were collected in each tube. Sensitivity threshold values were fitted when the concentration was below the sensitivity limits.

#### Behavioral Tests Before Mating

As shown in Figure 1, behavioral tests for F1 were performed from postnatal day 70 to 110.

##### Open Field Test

As described in previous studies (23, 24) and shown in Figure 3A, each mouse was individually placed in the center of a 45 cm × 45 cm × 20 cm (width × length × height) open-field chamber containing 16 × 16 crossed infrared beams set along the sides at intervals of 2.5 cm (Figure 3A; LE 8811, Panlab, S.L.U. Barcelona, Spain) (23). Locomotion was recorded for 30 min by tracking the position of the mouse and the number of times that beams were crossed. Data were analyzed using the Acti-Track program (Panlab, S.L.U.). The total distance traveled and time spent in the central area (23 cm × 23 cm in the middle of the field) was measured. Additionally, data were presented in three different times to define the onset time of anxiety-like-behavior.

##### ORT and Object-in-Location Recognition Test (OLT) in Rodents

As shown in Figure 4A, ORT/OLT were performed in a 30 cm × 30 cm × 39 cm white, rectangular open field as reported in previous studies (22, 25). Mice were exposed to the experimental apparatus in the open field for 15 min/day for 3 days to habituate them to the environment. The recognition tests (ORT/OLT) are consisted of a training phase and a test phase individually. During the training phase (5 min), two identical objects were placed in the field. After a 10-min retention interval, mice were placed back in the field to do the test phase (3 min). To examine object recognition memory, one of the two objects was replaced by a novel object in a test phase (ORT) (22, 25). After ORT session, we performed a training phase for OLT, and changed the location of one object in a test phase to examine object-in-location memory (OLT) (Figure 4B) (25). Previous studies showed mice tended to spend a longer time exploring the novel object/location if they remembered the previous object (22, 25). Mice were performed the tests in one-time trial. All sessions were recorded on video (Sony DVR 4 camera, recording resolutions: HD 352 × 480; 330,000-pixel, frame rates: 10 frames per second) and exploration time was measured manually with a stopwatch. The discrimination ratio was determined by dividing the time spent exploring the novel object by the total time exploring both objects during the test session.

#### Maternal Behavior Tests

##### Pup Retrieval Behavior

In order to assess maternal behavior in F1 mice, we first measured the length of time required to retrieve the pups that had been removed from the nest using a slightly modified protocol from a previous study (15). The test was performed during the dark phase of F1 at postnatal day 110. The experimental procedure was recorded with a video camera. Initially, dams and pups were briefly removed from their home cage. Then, three pups were placed in different corners of the cage, away from the nest. The dam was then reintroduced into the cage. Time of first pup identification was defined as the time that the dam took to touch a pup with her nose. Such behavior was followed by picking up the pup in her mouth to bring it back to the nest. The time required for first pup identification and for replacing all three pups in the nest was measured manually using a stopwatch. If the mice could not retrieve all pups within 15 min, the test was interrupted and remaining pups were replaced in the nest.

##### Nursing and Pup Licking

F1 dam–pup interaction was also measured by observing arched-back nursing and pup-licking behaviors (26). Time spent nursing was defined as the time that mice spent in the nest in an arched-back posture. Behaviors not related to dam–pup interaction (leaving the nest, eating, and drinking) were also measured. These behaviors were observed for two 1-h periods per day (in both the light phase, 9:00–10:00, and the dark phase, 1:00–2:00). The behavior of mice was recorded with a video camera, and time was measured manually with a stopwatch.

### Statistical Analysis

Statistical comparisons were performed by one- or two-way ANOVA followed by the Bonferroni *post hoc* test using SPSS Software version 22.0 (IBM SPSS, Armonk, NY, USA). Differences were considered significant at *p* < 0.05. All values are presented as the mean ± SEM.

To analyze the relationship between cognitive function and maternal behavior (Figure 7), the correlations between discrimination ratio of object recognition or object-in-location and maternal behavioral parameters were calculated using the correlation graph simple linear regression method by SPSS.

## Results

### The Effect of PTU Treatment on the Number of Pups Delivered

Propylthiouracil treatment did not induce failure of delivery or abandonment of offspring in F0 mice (data not shown), whereas PTU treatment during *in utero* and postnatal periods did induce such tendencies of F1 mice in dose-dependent manner (Table S1 in Supplementary Material). In F1, one mouse treated with 5 ppm PTU did not deliver pups at all, although the pregnancy was confirmed. Furthermore, two mice treated with 50 ppm PTU did not deliver and four mice abandoned their offspring. After excluding these mice, the number of delivered pups was counted (Table 1). There was no significant difference in the number of delivered mice in F0 groups, whereas in F1 mice, PTU treatment during *in utero* and postnatal periods significantly affected the number of pups delivered. While control mice delivered 6.25 ± 0.372 mice, mice treated with 5 or 50 ppm only delivered 4.87 ± 0.274 mice or 3.6 ± 0.267 mice, respectively (by Bonferroni test; *p* = 0.007, control vs. 5 ppm group; *p* = 0.008, control vs. 50 ppm group).

**Table 1:** Number of offspring from individual mothers.

Dam	Control			5 ppm			50 ppm		
			
	Male	Female	Total	Male	Female	Total	Male	Female	Total
**F0 Generation**									
F0-1	3	4	7	2	5	7	3	6	9
F0-2	2	4	6	1	6	7	1	4	5
F0-3	3	3	6	2	4	6	2	3	5
F0-4	4	5	9	2	3	5	1	2	3
F0-5	–	–	–	4	3	7	2	3	5
F0-6	–	–	–	0	2	2	3	5	8
F0-6	–	–	–	0	2	2	3	5	8
Average			6.67			5.67			5.83
SEM			0.494			0.802			0.901

**F1 Generation**									
F1-1	2	5	7	4	2	6	2	3	5
F1-2	3	4	7	3	3	6	2	1	3
F1-3	1	5	6	2	3	5	2	0	2
F1-4	2	3	5	2	2	4	1	3	4
F1-5	3	2	5	2	3	5	1	2	3
F1-6	4	2	6	3	4	7	2	2	4
F1-7	3	2	5	1	3	4	3	1	4
F1-8	3	5	8	2	2	4	2	2	4
F1-9	5	1	6	3	1	4	1	2	3
F1-10	3	6	9	2	3	5	1	3	4
F1-11	2	3	5	3	1	4	–	–	–
F1-12	4	2	6	4	2	6	–	–	–
F1-13	–	–	–	3	2	5	–	–	–
F1-14	–	–	–	1	4	5	–	–	–
F1-15	–	–	–	2	1	3	–	–	–
Average			6.25			4.87**			3.60***
SEM			0.372			0.274			0.267

**p < 0.01, ***p < 0.001.

*The failure number of F1 dams of giving birth and taking care of pups were omitted*.

### Change in TH Concentrations in F1 Mice

**Figure 2:**
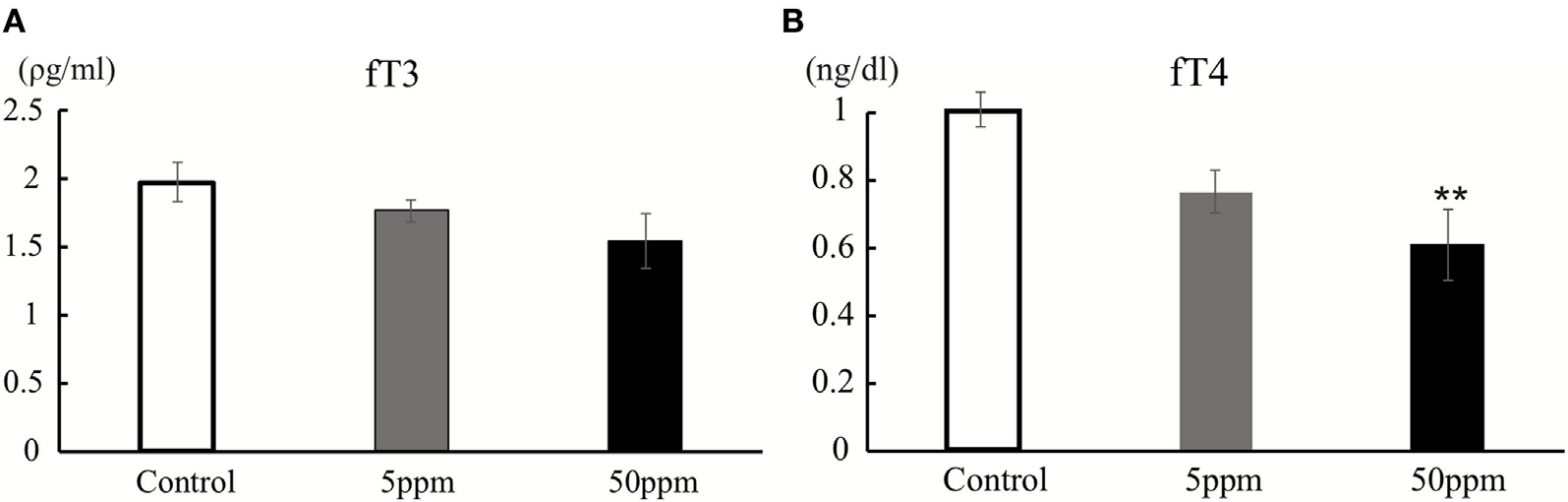
Thyroid hormone levels at postnatal day 70. Free T3 (fT3) **(A)** and free T4 (fT4) **(B)** were measured by CMIA (see text for details). There was a significant difference in fT4 levels between the control and 50 ppm group (Data are mean ± SEM, **p < 0.01, compared to the control group by Bonferroni test).

As shown in Figure 2, there was no significant difference in fT3 levels among F1 groups (by ANOVA *F*_(2,12)_ = 2.051; *p* = 0.171). However, fT4 levels were significantly altered by PTU treatment during *in utero* and postnatal periods in F1 mice [by ANOVA; *F*_(2,12)_ = 6.790; *p* = 0.011]. *Post hoc* analysis showed that fT4 levels in the 50 ppm group were lower than those of the control group (by Bonferroni test; *p* = 0.008), signifying PTU exposure leads to mild hypothyroidism in the 50 ppm group, even as late as postnatal day 70.

### The Effect of PTU Treatment During *in Utero* and Postnatal Periods on Locomotor Activity and Cognition

#### Changes in Locomotor Activity

**Figure 3:**
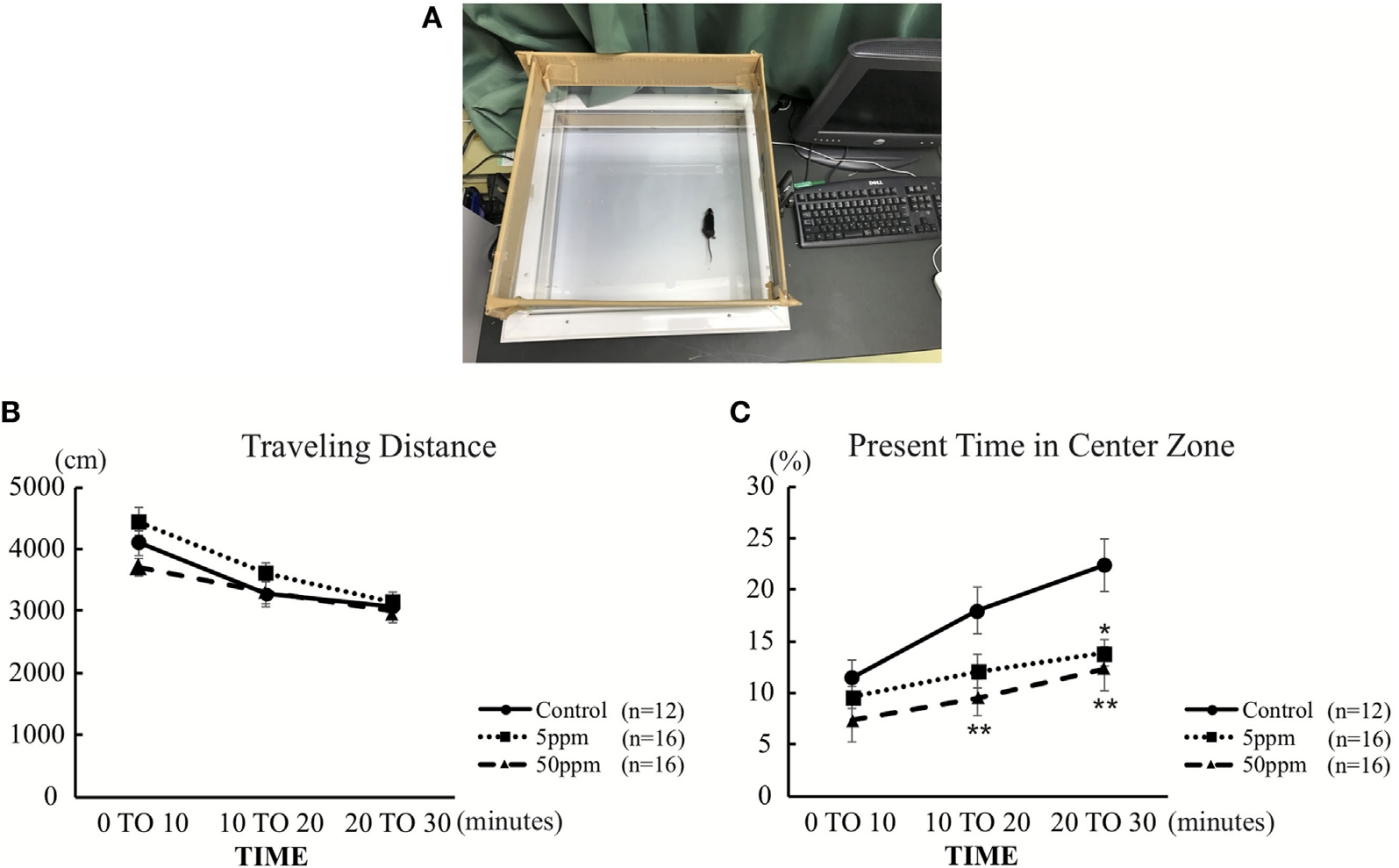
The effect of propylthiouracil treatment during *in utero* and postnatal periods in open field test. **(A)** Open field test apparatus. The open field was divided into two zones; peripheral area and central area. **(B)** Traveling distance in the open field. There was no difference in traveling distance among groups. **(C)** Time in the central area. The time was significantly altered at 20–30 min in the 5 ppm group and at 10–20 and 20–30 min in the 50 ppm group. Data are the mean ± SEM, *p < 0.05; **p < 0.01 compared to the control group by Bonferroni test.

We first performed the open field test on F1 mice. Activity in the open field was monitored for 30 min. As shown in Figure 3B, there was no significant difference in traveling distance among groups. However, as shown in Figure 3C, 5 ppm group spent less time in the central area over 20–30 min compared with the control group (by Bonferroni test; *p* = 0.032), whereas 50 ppm group spent less time in the central area over 10–20 and 20–30 min (by Bonferroni test; *p* = 0.010 at 10 min, *p* = 0.007 at 20 min). These results indicate an increase in anxiety induced by mild and moderate hypothyroidism without altering locomotor activity.

#### Aberrant Cognitive Function

**Figure 4:**
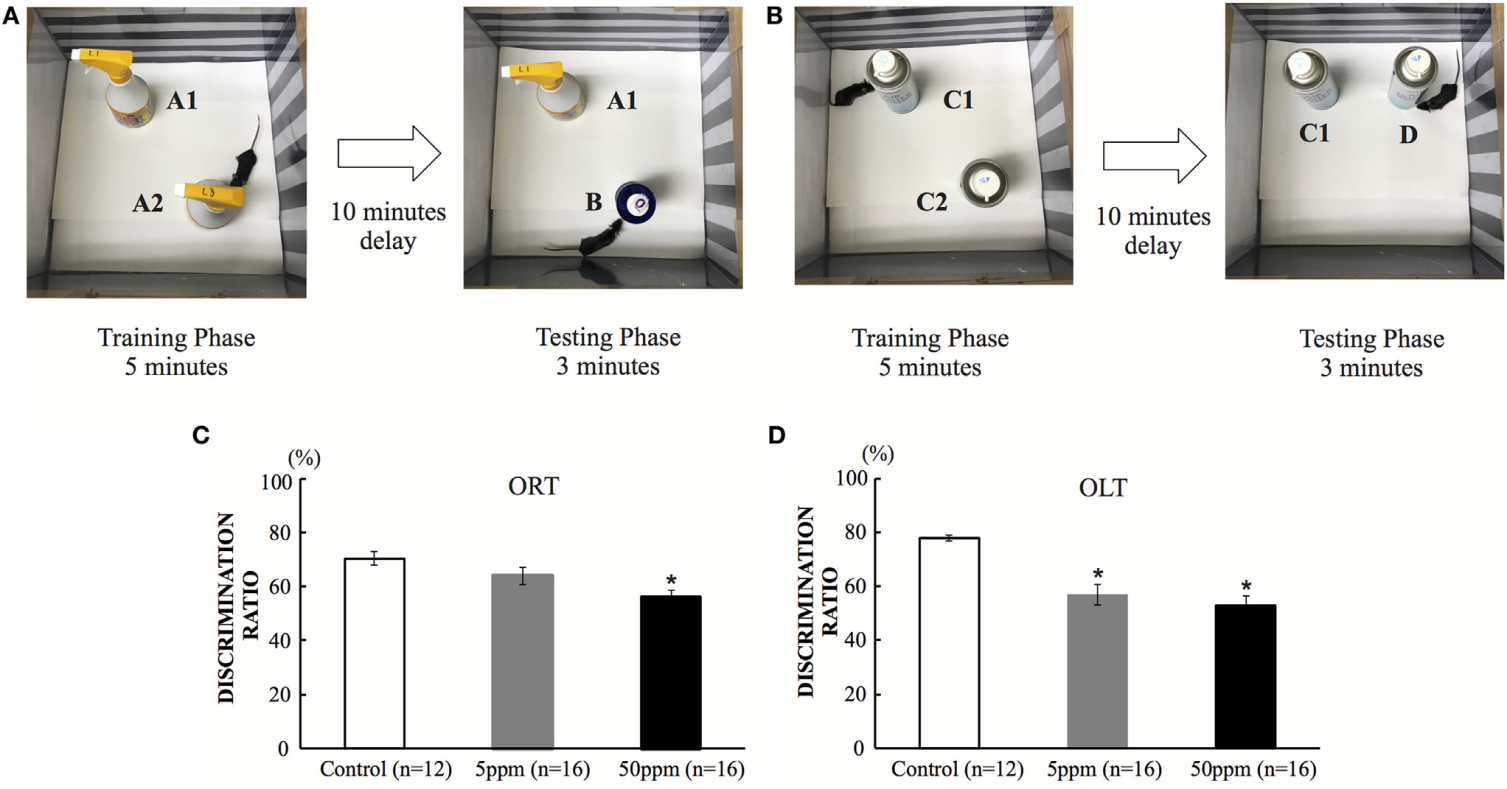
The effect of propylthiouracil treatment during *in utero* and postnatal periods in object recognition test (ORT) and OLT. **(A)** ORT apparatus. A mouse was put in the box and was introduced to two identical objects (Object A1 and A2). In the test phase, one object was replaced by a novel object (Object B). **(B)** OLT apparatus. A mouse was put in the box and was introduced to two identical objects (Object C1 and C2). In the test phase, one object was displaced to a new location (Object D). **(C)** Discrimination ratio of object recognition in ORT. The ratio decreased significantly in the 50 ppm group, indicating that the mice could not recognize the novel object. Data are the mean ± SEM, *p < 0.05 compared with the control group by the Bonferroni test. **(D)** Discrimination ratio of object-in-location in OLT. The discrimination ratio decreased significantly in the 5 and 50 ppm groups. Data are mean ± SEM, *p < 0.05 compared to the control group by Bonferroni test.

In the ORT, the discrimination ratio was significantly decreased in the 50 ppm group compared with the control group (by Bonferroni test; *p* = 0.042) (Figure 4C). On the other hand, the 5 ppm group did not show any significant difference compared with the control group. In the OLT, the discrimination ratio decreased significantly in both the 5 and 50 ppm groups compared with the control group (*p* = 0.035) (Figure 4D). These results indicate that mild hypothyroidism induces cognitive impairment. These results are essentially the same as those reported in previous studies (9, 27).

### Alteration of Maternal Behavior Induced by PTU Treatment During in Utero and Postnatal Periods

#### Disruption of Pup Retrieval

**Figure 5:**
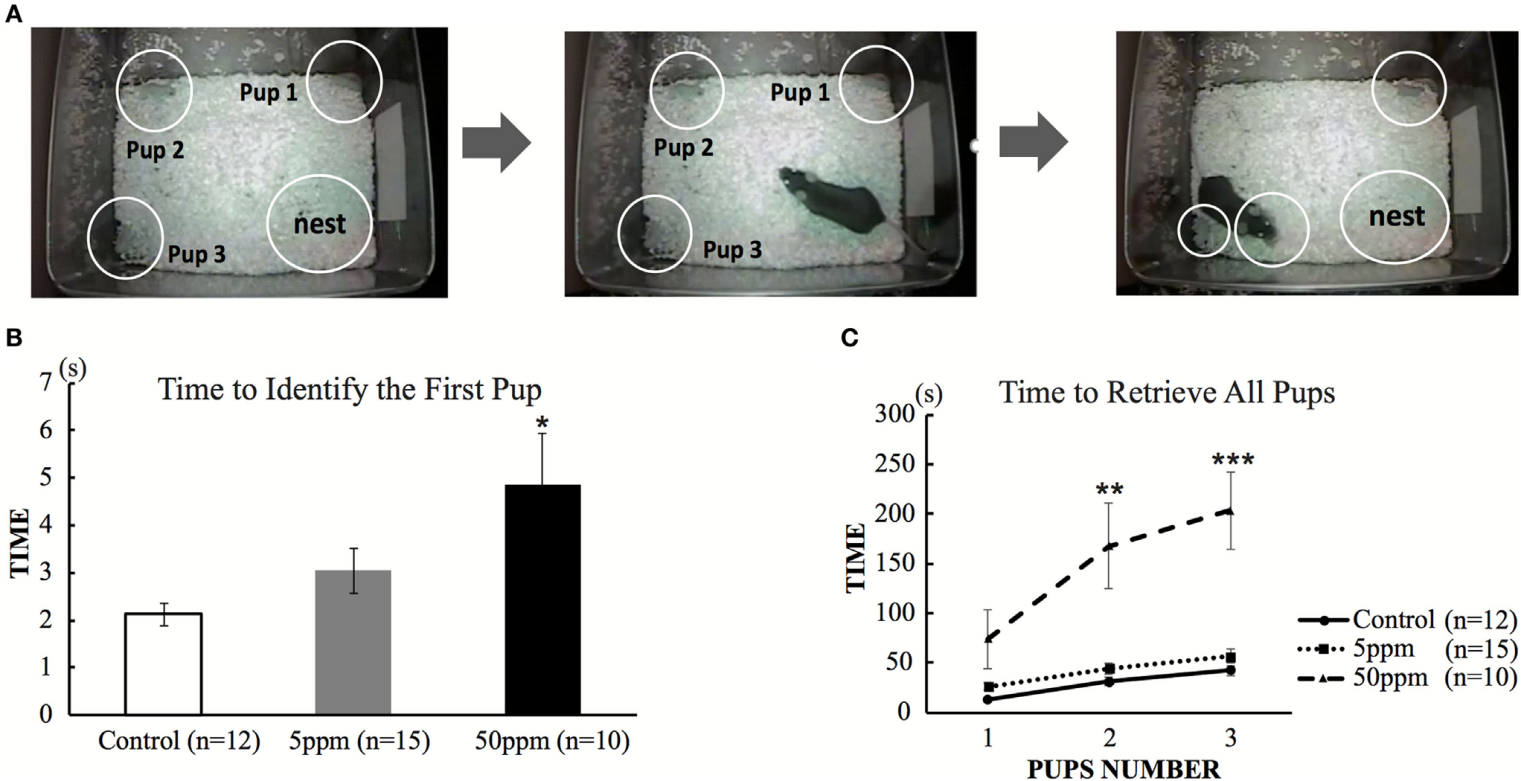
The effect of propylthiouracil treatment during *in utero* and postnatal periods on maternal behavior. **(A)** Pup retrieval test procedure. Three pups were placed in each corner followed by reintroduction of the dam into the cage. The time until the dam recognized the first pup by touching by it with nose and until the dam collected all three pups back in the nest were counted. **(B)** Time required to identify the first pup. The time was longer in mice from the 50 ppm group. **(C)** Time required to retrieve all three pups. The time was longer in mice from the 50 ppm group. Data are the mean ± SEM, *p < 0.05; **p < 0.01; ***p < 0.001 compared to the control group by ANOVA and Bonferroni test.

To examine the effect of hypothyroidism on maternal behavior, we first examined the pup retrieval behavior of F1 mice on postnatal day 110. Dams were transiently removed from the cage. Three pups were placed in a different corner of the cage, outside of the nest. Immediately after the dam was returned to the cage, the dam identified the first pup within a few seconds, picked it up and replaced in the nest. As shown in Figure 5B, ANOVA showed a significant treatment effect (*F*_(2, 34)_ = 6.482; *p* = 0.004), indicating that PTU treatment during *in utero* and postnatal periods altered the time required to identify the first pup. As shown in Figures 5B,C, mice in the 50 ppm group required a significantly longer time to identify the first pup (control mice 2.13 ± 0.235 s; 5 ppm 3.04 ± 0.472 s; 50 ppm, 4.89 ± 1.081 s; *p* = 0.028, by Bonferroni test). They also took longer to place all three pups back in the nest (by Bonferroni test; *p* = 0.0001 vs. control). Some mice in the 50 ppm group required longer than 15 min to finish retrieval. However, the time to finish retrieval in the 5 ppm group was not different from that of control mice.

#### Decrease in Nursing and Pup Licking

**Figure 6:**
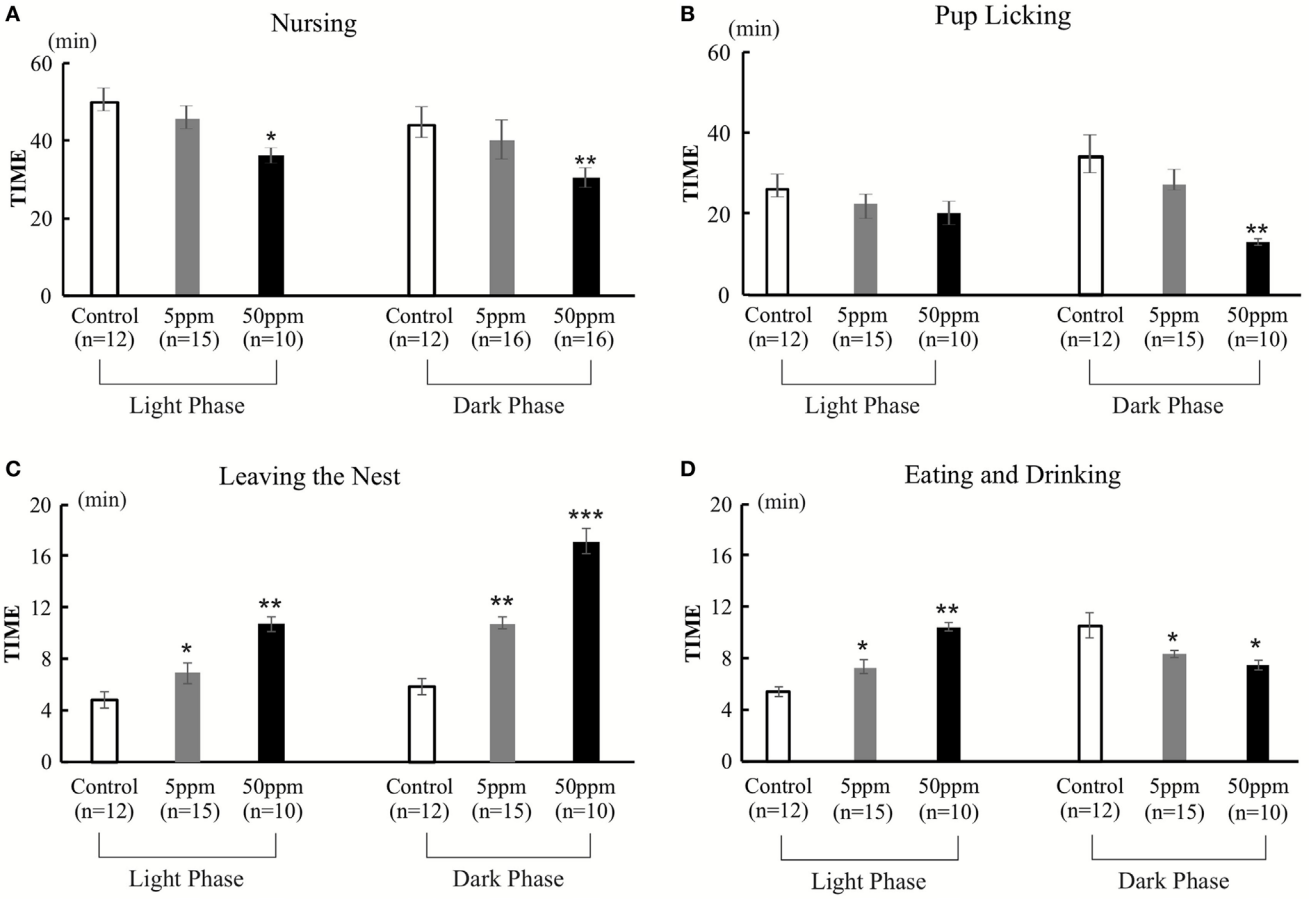
The duration of nursing, pup licking, leaving the nest, and eating and drinking in 1 h. Behavior was observed for two 1-h periods per day (light phase, 9:00–10:00; dark phase, 1:00–2:00). **(A)** Duration of nursing. Mice in the 50 ppm group spent less time nursing, both in light and dark phases. **(B)** Duration of pup licking. Mice in the 50 ppm group spent less time licking their pups in the dark phase. **(C)** Leaving the nest. Hypothyroid-treated mice spent longer out of the nest both in the light and dark phases **(D)** Duration of eating and drinking. Hypothyroid-treated mice spent more time eating and drinking in the light phase and less time in the dark phase. Data are the mean ± SEM, *p < 0.05; **p < 0.01; ***p < 0.001 compared with the control group by Bonferroni test.

We evaluated two aspects of F1 dams’ behavior on postnatal day 2. One aspect is related to mother–pup interactions, such as taking a nursing posture (arched-back nursing) in the nest and licking the pups. The other aspect examined behaviors not related to mother–pup interaction, such as time spent outside of the nest, and time spent eating and drinking. The behaviors were observed for 1 h, twice a day (in the light phase and in the dark phase). A significant treatment effect was observed by ANOVA for nursing behavior [light phase, *F*_(2, 34)_ = 152.973, *p* < 0.001; dark phase, *F*_(2,34)_ = 61.637, *p* = 0.0001], and pup licking [light phase, *F*_(2, 34)_ = 30.048, *p* = 0.0001; dark phase, *F*_(2,34)_ = 120.655, *p* = 0.0001], indicating that PTU treatment during *in utero* and postnatal periods disrupted behaviors related to mother–pup interaction (Figures 6A,B). *Post hoc* analyses revealed mice in the 50 ppm group showed significantly decreased nursing in both the light and dark phases (light phase, *p* = 0.037; dark phase, *p* = 0.003) and pup licking in the dark phase (*p* = 0.002), whereas no significant difference in 5 ppm group compared to control group. We also found that PTU treatment during *in utero* and postnatal periods altered the time spent outside of the nest in both groups [light phase, *F*_(2,34)_ = 91.519; 5 ppm, *p* = 0.035; and 50 ppm, *p* = 0.000. dark phase, *F*_(2,34)_ = 91.519; 5 ppm, *p* = 0.01 and 50 ppm, *p* = 0.0001] (Figure 6C). *Post hoc* analysis showed a significant increase in the time spent outside of the nest in both the 5 ppm (*p* = 0.028) and 50 ppm (*p* = 0.003) groups. Because the mouse is a nocturnal animal, they usually spend longer eating and drinking during the night (dark phase). Control mice showed a normal circadian rhythm by spending longer time eating and drinking during the dark phase, whereas PTU-treated mice lacked this circadian rhythm (Figure 6D). These results indicate that *in utero* and postnatal PTU-induced mild/moderate hypothyroidism disrupted maternal behavior.

### Correlation Between Discrimination Ratio of Object Recognition or Object-in-Location and Maternal Behaviors

**Figure 7:**
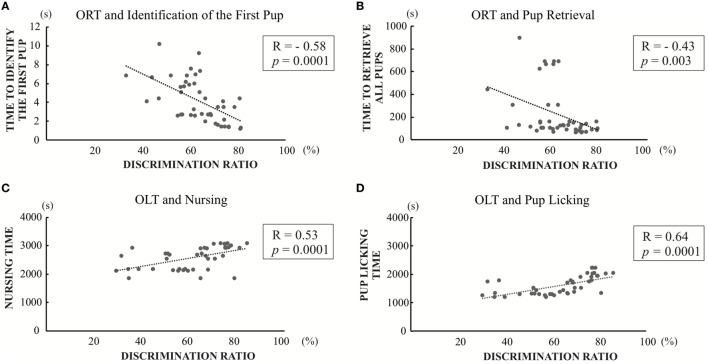
Significant correlation graph between discrimination ratios of object recognition test (ORT) or OLT and maternal behavior values. **(A)** Correlation between discrimination ratios in object recognition and first pup identification time (*R* = −0.58; *p* = 0.0001). **(B)** Correlation between discrimination ratios in object recognition and three pup retrieval time (*R* = −0.43; *p* = 0.003). **(C)** Correlation between discrimination ratios in object-in-location and nursing time (*R* = 0.53; *p* = 0.0001) **(D)** Correlation between discrimination ratios in object-in-location and pup-licking time (*R* = 0.64; *p* = 0.0001).

In light of previous findings showing maternal behavior is associated with cognitive function (26, 28), we correlated the maternal behavior parameters with the discrimination ratios for the memory function of Object Recognition or Object-in-Location measured in the present study. There were significant negative correlations between discrimination ratio of object recognition and first pup identification time (*R* = −0.58; *p* = 0.0001) (Figure 7A), as well as three pup retrieval time (*R* = −0.43; *p* = 0.003) (Figure 7B). There were no statistical significances between the discrimination ratio of object recognition and either nursing time or pups licking time (Figure S1 in Supplementary Material). In addition, there were significant positive correlations between the discrimination ratio of object-in-location and both nursing time and pup-licking time (*R* = 0.53; *p* = 0.0001 and *R* = 0.64; *p* = 0.0001) (Figures 7C,D). In contrast, no significant correlations were observed between the discrimination ratio of object-in-location and both time required for identification of the first pup and three-pup retrieval time (Figure S1 in Supplementary Material). There was no significant correlation between open field test and any of the maternal behavioral parameters (data not shown). Overall, these results indicate that cognitive impairment caused by PTU treatment during *in utero* and postnatal periods may be partly involved in generating aberrant maternal behavior.

## Discussion

In the present study, we examined the effect of PTU treatment during *in utero* and postnatal periods on maternal behavior in mice. We measured THs of F1 mice in adulthood and found a PTU dose-dependent weak decrease in fT4 levels, indicating that, although hypothyroidism during brain development may be mainly responsible for altering behavior, a continuous decrease in TH levels may have also contributed to behavioral alteration. As reported previously in male rats (29), *in utero* and postnatal PTU-induced mild/moderate hypothyroidism increased anxiety (Figure 3), impaired cognitive function (Figure 4), disrupted maternal behavior, such as increased time to identify and retrieve pups (Figure 5), decreased time for nursing and pup licking, increased time for being away from the nest, and disrupted circadian rhythm in eating and drinking (Figure 6). The magnitude of cognitive deficit was correlated with the level of observed aberrant maternal behavior (Figure 7). Moreover, we found PTU treatment during *in utero* and postnatal periods significantly affected the number of F1 offspring. These results, therefore, indicate that *in utero* and postnatal PTU-induced mild/moderate hypothyroidism disrupted maternal behavior at least in part through impaired cognitive function.

Previous studies have shown that low dose (1 ppm) PTU treatment during *in utero* and postnatal periods in rats caused a decrease in total T4 levels, and 3 ppm treatment elevated thyroid-stimulating hormone levels in postnatal day 14 (30) or postnatal day 21 (31), but not in postnatal day 90. Another study in rats showed that 3 ppm PTU treatment did not alter total T3 (tT3) on postnatal day 3, but decreased on postnatal day 15, whereas tT3 returned to be normal on postnatal day 22 (6). In the present study, we also measured fT3 and fT4 levels to investigate whether the exposure of PTU treatment during *in utero* and postnatal periods caused the changes of the TH status at postnatal day 70. We found both fT3 and fT4 levels on 5 ppm-treated group tend to decrease, even though not statistically significant. On the other hand, 50 ppm-treated group showed low TH levels (Figure 2). Based on these results, we categorized 5 ppm group as “mild” and the 50 ppm group as “moderate” hypothyroid mice model. Although the mechanisms causing this continuous decrease in TH levels are not yet clear, PTU treatment during *in utero* and postnatal periods may have permanently disrupted the function of the thyroid gland, as well as exposed the pups to lower levels of maternal THs.

Thyroid hormones have direct effects on granulosa cells, luteal cells, and oocytes, indicating a direct interference with normal ovarian function in animal (32) and human cases (33, 34). Therefore, hypothyroid condition may cause disruption of reproductive function and development (35–37). In animal study, abnormality of THs level impaired the development of ovarian cells and caused infertility (32). In this study, we found that 50 ppm F1 generation showed lower number of pups (Table 1). This result indicates *in utero* and postnatal PTU exposure may disrupt placental and ovarian development and consequently decrease fecundity.

In this study, we evaluated the maternal behavior of F1 offspring, and found that the behaviors were affected by PTU exposed during *in utero* and postnatal periods (Figures 5 and 6). Responses or behaviors shown by mammalian females are important for the physiological and behavioral development of their offspring (38). A study of an *in utero* maternal process showed that the fetal stage is a crucial period for behavioral development (15). In rodents, maternal behaviors are exhibited in several ways, including pup retrieval, licking, nursing, and crouching over pups to keep them warm (39). The expression of maternal behaviors during gestation and postpartum period is regulated by dynamic changes in hormonal secretion (39, 40), such as prolactin, that can affect the nurturing behaviors of both mothers and their female offspring (15). Prolactin acts in the medial preoptic area (39, 41) to promote the activation of neural circuitry that is associated with nurturing behavior in adulthood. On the other hand, variations in maternal licking/grooming and arched-back nursing have been associated with the development of neural systems that mediate hypothalamic–pituitary–adrenal and behavioral responses to stress as well as certain forms of learning and memory (20, 26, 42). Furthermore, multiple sensory inputs such as visual, auditory, olfactory, and tactile stimuli trigger maternal behavior (43). According to a study in rats, perinatal hypothyroidism results in irreversible damage to auditory function resulting in hearing loss (44); consequently, F1 mothers may not be able to hear ultrasonic vocalization or calls produced by pups that should have immediately induced retrieval (45). Because THs are responsible for the development of various neuronal and neuroendocrinological functions, PTU treatment during *in utero* and postnatal periods may have disrupted functional development of such systems even if it is mild or moderate.

In the open field test, we found no significant difference in traveling distance among groups (Figure 3A), although the decreased time spent in the center zone by the PTU-treated group may indicate an increase in anxiety (Figure 3B). Increased anxiety due to *in utero* and postnatal hypothyroidism (9) and in thyroid receptor knockout mouse models (11) has been reported previously. Moreover, previous studies have shown that TH manipulation in early life affects hippocampal glucocorticoid receptor protein levels (20, 46). In the present study, we also found that PTU treatment during *in utero* and postnatal periods induced anxiety-like behavior in female mice. Thus, increased anxiety may alter maternal behavior.

We evaluated the disruption of cognitive function in mice by conducting Object Recognition and Object-in-Location Tests. Previous studies have shown that developmental TH insufficiency impairs spatial learning memory in rodent models (29). Our results showed that the discrimination ratio of object recognition of the 50 ppm group decreased significantly (Figure 4C) and considered to be influenced by either the hippocampal or cortical lesions (47). Among such brain regions, the perirhinal cortex may play a major role in controlling object recognition memory (48). When this region is damaged, object recognition performance is impaired (49). On the other hand, the result for OLT showed that the discrimination ratio of object-in-location in both PTU-treated groups decreased significantly (Figure 4D), suggesting that the mice may not be able to discriminate the object in the new location due to decreased spatial memory acquisition (50). Such impairments may be caused by aberrant function of the hippocampus (51, 52). Previous studies have shown that maternal care is associated with spatial learning and memory (26, 28). This possibility has been confirmed further in the present study, showing correlations between discrimination ratio of object recognition or object-in-location and maternal behavior values (Figure 7). Although additional analysis may be required, *in utero* and postnatal PTU-induced mild/moderate hypothyroidism disrupted maternal behavior, which may be caused in part by impairment of cognition and anxiety.

We previously reported the effects of PTU administration on the TH status and cognitive function in male mouse offspring, using the same administration schedule as for the present study (either 5 ppm or 50 ppm) (22). Unlike the rat model, which showed a significant reduction of serum T3 and T4 levels and growth retardation by 10 ppm PTU, and decreased T3 levels with increased TSH levels by 3 ppm (6), our mouse model with 5 ppm did not show any increase in TSH levels at postpartum day 21 in both dams and pups. Instead, only a weak decrease in fT4 levels was observed only in dams by 5 ppm PTU. With 50 ppm treatment, on the other hand, although dams showed an elevation of TSH levels with decreased fT3 and fT4 levels, pups showed only a weak decrease in fT4 levels without a corresponding increase in TSH levels. Despite such a small change in TH status, offspring of both groups showed a disrupted cognitive function in a PTU dose-dependent manner in both male (22) and female (present study) offspring. Furthermore, 50 ppm-treated offspring showed decreased levels of NMDA receptor subunits in the hippocampus, whereas such decrease was not observed in 5 ppm-treated group. Taken together with these findings, we defined 5 ppm PTU group as “mild” and 50 ppm group as “moderate.” However, these definitions may be rather subjective as we do not have clear criteria to discriminate among mild, moderate, and severe hypothyroidism in mouse model. Further study with finer titration of PTU dose may be required to clarify such difference. Nevertheless, our present study clearly demonstrated that gestational and postpartum PTU treatment that induced only a little alteration of TH status in both dams and pups caused an alteration of maternal behavior of offspring, indicating an important role of TH on regulating such behavior.

There are some limitations in this study. First, although the decrease in TH levels in PTU-treated F1 mice at postnatal day 70 was observed, it is not clear whether the behavioral alterations seen in F1 rodents are due to prenatal or postnatal hypothyroidism or to maternal hypothyroidism during gestation. TH may regulate target gene expression differently during prenatal and postnatal period. Maternal hypothyroidism may alter the expression of maternal factor that may play an important role in fetal development. Second, our behavioral results indicated that the effect of hypothyroidism is different among the brain functions and some of them are critically affected even under small TH changes. However, no report has shown the lowest PTU dose that causes adverse effect. To clarify these issues, additional study including gene expression analysis is required.

In summary, mild or moderate hypothyroidism due to PTU treatment during *in utero* and postnatal periods were seen to disrupt maternal behavior by inducing anxiety and memory impairment. Additionally, we found that a decrease in fecundity of offspring may be caused by abnormal TH status. Our series of studies have provided useful information for further understanding the role of THs on the construction in pregnancy and mother–offspring interactions. More careful attention may be required to support hypothyroidism patients when they want to have children, even if their TH status is within normal ranges. Furthermore, precise control of thyroid function during pregnancy and the lactating periods may be important for both maternal care and cognitive function development in offspring.

## Ethics Statement

The animal experimentation protocol in this study was approved by the Animal Care and Experimentation Committee of Gunma University. All efforts were made to minimize the number of animals used in this study and their suffering.

## Author Contributions

MAK, YT, and IA conducted the complete experiment and prepared the data and manuscript. WM and NK had responsibility for the whole experiment, earned the grant, made the strategy, and prepared the manuscript. AH and MK contributed by experimental support.

## Conflict of Interest Statement

The authors declare that the research was conducted in the absence of any commercial or financial relationships that could be construed as a potential conflict of interest.

## Supplementary Material

Figure S1 Insignificant correlation graph between discrimination ratios of object recognition or object-in-location and maternal behavior values. **(A)** Correlation between discrimination ratios of object recognition and nursing time (*R* = 0.28). **(B)** Correlation between discrimination ratios of object recognition and pup licking (*R* = 0.36). **(C)** Correlation between discrimination ratios of object-in-location and identification of the first pup (*R* = −0.25). **(D)** Correlation between discrimination ratios of object-in-location and three pup retrieval time (*R* = −0.38).

## References

[1] Koibuchi N Jingu H Iwasaki T Chin WW. Current perspectives on the role of thyroid hormone in growth and development of cerebellum. Cerebellum (2003) 2:279–89. 10.1080/14734220310011920 14964687

[2] Hasebe M Matsumoto I Imagawa T Uehara M. Effects of an anti-thyroid drug, methimazole, administration to rat dams on the cerebellar cortex development in their pups. Int J Dev Neurosci (2008) 26:409–14. 10.1016/j.ijdevneu.2008.03.007 18456449

[3] Shimokawa N Yousefi B Morioka S Yamaguchi S Ohsawa A Hayashi H Altered cerebellum development and dopamine distribution in a rat genetic model with congenital hypothyroidism. J Neuroendocrinol (2014) 26:164–75. 10.1111/jne.12135 24460919

[4] Rami A Patel AJ Rabie A. Thyroid hormone and development of the rat hippocampus: morphological alterations in granule and pyramidal cells. Neuroscience (1986) 19:1217–26. 10.1016/0306-4522(86)90135-1 3822116

[5] Rami A Rabie A Patel AJ. Thyroid hormone and development of the rat hippocampus: cell acquisition in the dentate gyrus. Neuroscience (1986) 19:1206–16. 10.1016/0306-4522(86)90134-X 3822115

[6] Gilbert ME. Impact of low-level thyroid hormone disruption induced by propylthiouracil on brain development and function. Toxicol Sci (2011) 124:432–45. 10.1093/toxsci/kfr244 21964421

[7] Bocco BM Werneck-de-Castro JP Oliveira KC Fernandes GW Fonseca TL Nascimento BPP Type 2 deiodinase disruption in astrocytes results in anxiety-depressive-like behavior in male mice. Endocrinology (2016) 157:3682–95. 10.1210/en.2016-1272 27501182 PMC5007895

[8] Buras A Battle L Landers E Nguyen T Vasudevan N. Thyroid hormones regulate anxiety in the male mouse. Horm Behav (2014) 65:88–96. 10.1016/j.yhbeh.2013.11.008 24333846

[9] Darbra S Garau A Balada F Sala J Martí-Carbonell MA. Perinatal hypothyroidism effects on neuromotor competence, novelty-directed exploratory and anxiety-related behaviour and learning in rats. Behav Brain Res (2003) 143:209–15. 10.1016/S0166-4328(03)00041-X 12900047

[10] Yu L Iwasaki T Xu M Lesmana R Xiong Y Shimokawa N Aberrant cerebellar development of transgenic mice expressing dominant-negative thyroid hormone receptor in cerebellar Purkinje cells. Endocrinology (2015) 156:1565–76. 10.1210/en.2014-1079 25603044

[11] Venero C Guadano-Ferraz A Herrero AI Nordstrom K Manzano J De Escobar GM Anxiety, memory impairment, and locomotor dysfunction caused by a mutant thyroid hormone receptor alpha1 can be ameliorated by T3 treatment. Genes Dev (2005) 19:2152–63. 10.1101/gad.346105 16131613 PMC1221886

[12] Stohn JP Martinez ME Zafer M López-Espíndola D Keyes LM Hernandez A Increased aggression and lack of maternal behavior in Dio3-deficient mice are associated with abnormalities in oxytocin and vasopressin systems. Genes Brain Behav (2017) 3:1–13. 10.1111/gbb.12400 PMC5771999 28715127

[13] Murakami G. Distinct effects of Estrogen on mouse maternal behavior: the contribution of estrogen synthesis in the brain. PLoS One (2016) 11:e0150728. 10.1371/journal.pone.0150728 27007402 PMC4805179

[14] Ribeiro AC Musatov S Shteyler A Simanduyev S Arrieta-Cruz I Ogawa S siRNA silencing of estrogen receptor-expression specifically in medial preoptic area neurons abolishes maternal care in female mice. Proc Natl Acad Sci U S A (2012) 109:16324–9. 10.1073/pnas.1214094109 22988120 PMC3479618

[15] Sairenji T Ikezawa J Kaneko R Masuda S Uchida K Takanashi Y Maternal prolactin during late pregnancy is important in generating nurturing behavior in the offspring. Proc Natl Acad Sci U S A (2017) 114(49):13042–7. 10.1073/pnas.1621196114 29158391 PMC5724246

[16] Champagne FA Diorio J Sharma S Meaney MJ. Naturally occurring variations in maternal behavior in the rat are associated with differences in estrogen-inducible central oxytocin receptors. Proc Natl Acad Sci U S A (2001) 98:12736–41. 10.1073/pnas.221224598 11606726 PMC60123

[17] Stolzenberg DS Champagne FA. Hormonal and non-hormonal bases of maternal behavior: the role of experience and epigenetic mechanisms. Horm Behav (2016) 77:204–10. 10.1016/j.yhbeh.2015.07.005 26172856

[18] Champagne FA Francis DD Mar A Meaney MJ. Variations in maternal care in the rat as a mediating influence for the effects of environment on development. Physiol Behav (2003) 79:359–71. 10.1016/S0031-9384(03)00149-5 12954431

[19] Franks B Curley JP Champagne FA Mood and anxiety related phenotypes in mice. Neuromethods (2009) 42:1–20. 10.1007/978-1-60761-303-9

[20] Meaney MJ Diorio J Francis D Weaver S Yau J Chapman K Postnatal handling increases the expression of cAMP-inducible transcription factors in the rat hippocampus: the effects of thyroid hormones and serotonin. J Neurosci (2000) 20:3926–35. 10.1523/JNEUROSCI.20-10-03926.2000 10804232 PMC6772700

[21] Hellstrom IC Dhir SK Diorio JC Meaney MJ. Maternal licking regulates hippocampal glucocorticoid receptor transcription through a thyroid hormone-serotonin-NGFI-A signalling cascade. Philos Trans R Soc B Biol Sci (2012) 367:2495–510. 10.1098/rstb.2012.0223 22826348 PMC3405683

[22] Amano I Takatsuru Y Khairinisa MA Kokubo M Haijima A Koibuchi N. Effects of mild perinatal hypothyroidism on cognitive function of adult male offspring. Endocrinology (2018) 159:1910–21. 10.1210/en.2017-03125 29522169

[23] Amano I Takatsuru Y Toya S Haijima A Iwasaki T Grasberger H Aberrant cerebellar development in mice lacking dual oxidase maturation factors. Thyroid (2016) 26:741–52. 10.1089/thy.2015.0034 26914863 PMC4860669

[24] Khairinisa MA Takatsuru Y Amano I Erdene K Nakajima T Kameo S The effect of perinatal gadolinium-based contrast agents on adult mice behavior. Invest Radiol (2018) 53:110–8. 10.1097/RLI.0000000000000417 28915162

[25] Ennaceur A. One-trial object recognition in rats and mice: methodological and theoretical issues. Behav Brain Res (2010) 215:244–54. 10.1016/j.bbr.2009.12.036 20060020

[26] Liu D Diorio J Day JC Francis DD Meaney MJ. Maternal care, hippocampal synaptogenesis and cognitive development in rats. Nat Neurosci (2000) 3:799–806. 10.1038/77702 10903573

[27] van Wijk N Rijntjes E van de Heijning BJM. Perinatal and chronic hypothyroidism impair behavioural development in male and female rats. Exp Physiol (2008) 93:1199–209. 10.1113/expphysiol.2008.042416 18567604

[28] Champagne DL Bagot RC van Hasselt F Ramakers G Meaney MJ de Kloet ER Maternal care and hippocampal plasticity: evidence for experience-dependent structural plasticity, altered synaptic functioning, and differential responsiveness to glucocorticoids and stress. J Neurosci (2008) 28:6037–45. 10.1523/JNEUROSCI.0526-08.2008 18524909 PMC6670331

[29] Akaike M Kato N Ohno H Kobayashi T. Hyperactivity and spatial maze learning impairment of adult rats with temporary neonatal hypothyroidism. Neurotoxicol Teratol (1991) 13:317–22. 10.1016/0892-0362(91)90077-A 1886541

[30] Gilbert ME Sanchez-Huerta K Wood C. Mild thyroid hormone insufficiency during development compromises activity-dependent neuroplasticity in the hippocampus of adult male rats. Endocrinology (2016) 157:774–87. 10.1210/en.2015-1643 26606422

[31] Sui L Gilbert ME. Pre- and postnatal propylthiouracil-induced hypothyroidism impairs synaptic transmission and plasticity in area CA1 of the neonatal rat hippocampus. Endocrinology (2003) 144:4195–203. 10.1210/en.2003-0395 12933695

[32] Fedail JS Zheng K Wei Q Kong L Shi F. Roles of thyroid hormones in follicular development in the ovary of neonatal and immature rats. Endocrine (2014) 46:594–604. 10.1007/s12020-013-0092-y 24254997

[33] Ohara N Tsujino T Maruo T. The role of thyroid hormone in trophoblast function, early pregnancy maintenance, and fetal neurodevelopment. J Obstet Gynaecol Can (2004) 26:982–90. 10.1016/S1701-2163(16)30420-0 15560861

[34] Wakim A Polizoto S Buffo M Marrero M Burhold D Thyroid hormones in human follicular fluid and thyroid hormone receptors in human granulosa cells. Fertil Steril (1993) 59:1187–90. 10.1016/S0015-0282(16)55974-3 8495763

[35] Vanderpump MPJ Tunbrldge WMG Frencht JM Appietont D Bates D Clark F The incidence of thyroid disorders in the community: a twenty-year follow-up of the Whickham Survey. Clin Endocrinol (Oxf) (1995) 43:55–68. 10.1111/j.1365-2265.1995.tb01894.x 7641412

[36] Poppe K Velkeniers B Female infertility and the thyroid. Best Pract Res Clin Endocrinol Metab (2004) 18:153–65. 10.1016/j.beem.2004.03.004 15157833

[37] Poppe K Glinoer D Thyroid autoimmunity and hypothyroidism before and during pregnancy. Hum Reprod Update (2003) 9:149–61. 10.1093/humupd/dmg012 12751777

[38] Gonzalez A Lovic V Ward GR Wainwright PE Fleming AS. Intergenerational effects of complete maternal deprivation and replacement stimulation on maternal behavior and emotionality in female rats. Dev Psychobiol (2001) 38:11–32. 10.1002/1098-2302(2001)38:1<11::AID-DEV2>3.0.CO;2-B 11150058

[39] Bridges RS. Neuroendocrine regulation of maternal behavior. Front Neuroendocrinol (2015) 36:178–96. 10.1016/j.yfrne.2014.11.007 25500107 PMC4342279

[40] Rilling JK Young LJ. The biology of mammalian parenting and its effect on offspring social development. Science (2014) 345:771–6. 10.1126/science.1252723 25124431 PMC4306567

[41] Lucas BK Ormandy CJ Binart N Bridges RS Kelly PA. Null mutation of the prolactin receptor gene produces a defect in maternal behavior. Endocrinology (1998) 139:4102–7. 10.1210/endo.139.10.6243 9751488

[42] Caldji C Tannenbaum B Sharma S Francis D Plotsky PM Meaney MJ. Maternal care during infancy regulates the development of neural systems mediating the expression of fearfulness in the rat. Proc Natl Acad Sci U S A (1998) 95:5335–40. 10.1073/pnas.95.9.5335 9560276 PMC20261

[43] Dulac C Connell LAO Wu Z Neural control of maternal and paternal behaviors. Science (2014) 345:765–70. 10.1126/science.1253291 25124430 PMC4230532

[44] Wada H Yumoto S Iso H Irreversible damages to auditory system functions caused by perinatal hypothyroidism in rats. Neurotoxicol Teratol (2013) 37:18–22. 10.1016/j.ntt.2013.02.006 23422508

[45] Hahn ME Lavooy MJ. A review of the methods of studies on infant ultrasound production and maternal retrieval in small rodents. Behav Genet (2005) 35:31–52. 10.1007/s10519-004-0854-7 15674531

[46] Meaney MJ Aitken DH Sapolsky RM. Thyroid hormones influence the development of hippocampal glucocorticoid receptors in the rat: a mechanism for the effects of postnatal handling on the development of the adrenocortical stress response. Neuroendocrinology (1987) 45:278–83. 10.1159/000124741 3574606

[47] Buckmaster C Eichenbaum H Amaral D Suzuki W Rapp PR. Entorhinal cortex lesions disrupt the relational organization of memory in monkeys. J Neurosci (2004) 24:9811–25. 10.1523/JNEUROSCI.1532-04.2004 15525766 PMC6730224

[48] Aggleton JP Albasser MM Aggleton DJ Poirier GL Pearce JM. Lesions of the rat perirhinal cortex spare the acquisition of a complex configural visual discrimination yet impair object recognition. Behav Neurosci (2010) 124:55–68. 10.1037/a0018320 20141280 PMC2834571

[49] Albasser MM Davies M Futter J Aggleton JP. Magnitude of the object recognition deficit associated with perirhinal cortex damage in rats: effects of varying the lesion extent and the duration of the sample period. Behav Neurosci (2009) 123(1):115–24. 10.1037/a0013829 19170436

[50] Murai T Okuda S Tanaka T Ohta H. Characteristics of object location memory in mice: behavioral and pharmacological studies. Physiol Behav (2007) 90:116–24. 10.1016/j.physbeh.2006.09.013 17049363

[51] Barker GRI Warburton EC. When is the hippocampus involved in recognition memory? J Neurosci (2011) 31:10721–31. 10.1523/JNEUROSCI.6413-10.2011 21775615 PMC6622630

[52] Barker GRI Bird F Alexander V Warburton EC. Recognition memory for objects, place, and temporal order: a disconnection analysis of the role of the medial prefrontal cortex and perirhinal cortex. J Neurosci (2007) 27:2948–57. 10.1523/JNEUROSCI.5289-06.2007 17360918 PMC6672574

