# Supplementary material for: In Utero and Postnatal Propylthiouracil-Induced Mild Hypothyroidism Impairs Maternal Behavior in Mice
Source: Front Endocrinol (Lausanne). 2018 May 14;9:228. doi: 10.3389/fendo.2018.00228 (PMC5960672; doi:10.3389/fendo.2018.00228)
Supplement: Supplementary file 2 [file table_1.DOCX]

Supplemental Table 1. Failure in the process of giving birth and caring the offspring

| Group | Mother death | Failure of giving birth | Failure of caring the offspring |
| --- | --- | --- | --- |
| Control | 0/12 | 0/12 | 0/12 |
| 5ppm | 0/16 | 0/16 | 1/16 |
| 50ppm | 0/16 | 2/16 | 4/14 |
