# Supplementary material for: In Utero and Postnatal Propylthiouracil-Induced Mild Hypothyroidism Impairs Maternal Behavior in Mice
Source: Front Endocrinol (Lausanne). 2018 May 14;9:228. doi: 10.3389/fendo.2018.00228 (PMC5960672; doi:10.3389/fendo.2018.00228)
Supplement: Supplementary file 3 [file table_2.DOCX]

Supplemental Table 2. The number of samples in each experiment

| Experiment | Control | 5 ppm | 50 ppm |
| --- | --- | --- | --- |
| TH measurement | 10 | 10 | 10 |
| Behavior analysis | 12 | 16 | 16 |
| Total | 22 | 26 | 26 |
